# Liver fibrosis severity in chronic hepatitis B is associated with area-level rather than individual socioeconomic disadvantage: a nationwide multicentre study in Türkiye

**DOI:** 10.1186/s40249-026-01497-8

**Published:** 2026-09-03

**Authors:** Yeliz Çiçek, Şafak Kaya Kavak, Mustafa Kemal Çelen, Seyit Ali Büyüktuna, Mehmet Çelik, Burak Sarıkaya, Selda Aslan, Ahmet Şahin, Alper Tahmaz, Ahmet Melih Şahin, Fatma Kesmez Can, Caner Öksüz, Cihad Baysal, Mihrişah Aytekin, Sezin Hoşgel Sevdimbaş, Emre Bayhan, Melike Nur Özçelik, Hakan Sakin, Kazım Kıratlı, Merve Kılıç Tekin, Eda Çetin, Selcen Özer Kökkızıl, Pınar Çakmak, Gülten Ünlü, Ferah Öğüt, Rıdvan Dumlu, Ali Mert, Ercan Yenilmez

**Affiliations:** 1 Epidemiology Doctorate Program, Graduate School of Health Sciences, Istanbul Medipol University, Istanbul, Türkiye; 2 Department of Infectious Diseases and Clinical Microbiology, Gazi Yaşargil Training and Research Hospital, University of Health Sciences, Diyarbakır, Türkiye; 3 Department of Infectious Diseases and Clinical Microbiology, Faculty of Medicine, Dicle University, Diyarbakır, Türkiye; 4 Department of Infectious Diseases and Clinical Microbiology, Faculty of Medicine, Sivas Cumhuriyet University, Sivas, Türkiye; 5 Department of Infectious Diseases and Clinical Microbiology, Faculty of Medicine, Harran University, Şanlıurfa, Türkiye; 6 Department of Infectious Diseases and Clinical Microbiology, Sultan 2. Abdulhamid Han Training and Research Hospital, University of Health Sciences, Istanbul, Türkiye; 7 Department of Infectious Diseases and Clinical Microbiology, Gaziantep Islam Science and Technology University, Gaziantep, Türkiye; 8 Department of Infectious Diseases and Clinical Microbiology, Antalya Training and Research Hospital, University of Health Sciences, Antalya, Türkiye; 9 Department of Infectious Diseases and Clinical Microbiology, Faculty of Medicine, Giresun University, Giresun, Türkiye; 10 Department of Infectious Diseases and Clinical Microbiology, Faculty of Medicine, Atatürk University, Erzurum, Türkiye; 11 Department of Infectious Diseases and Clinical Microbiology, Şehit Aydoğan Aydın State Hospital, Şırnak, Türkiye; 12 Department of Infectious Diseases and Clinical Microbiology, Fatih Sultan Mehmet Training and Research Hospital, Istanbul, Türkiye; 13 Department of Infectious Diseases and Clinical Microbiology, Adana City Training and Research Hospital, Adana, Türkiye; 14 Department of Infectious Diseases and Clinical Microbiology, Gebze Fatih State Hospital, Gebze, Kocaeli, Türkiye; 15 Department of Infectious Diseases and Clinical Microbiology, Faculty of Medicine, Tekirdağ Namık Kemal University, Tekirdağ, Türkiye; 16 Department of Infectious Diseases and Clinical Microbiology, Faculty of Medicine, Dokuz Eylül University, İzmir, Türkiye; 17 Department of Infectious Diseases and Clinical Microbiology, Haseki Training and Research Hospital, Istanbul, Türkiye; 18 Department of Infectious Diseases and Clinical Microbiology, Mehmet Akif İnan Training and Research Hospital, Şanlıurfa, Türkiye; 19 Department of Infectious Diseases and Clinical Microbiology, Manisa City Hospital, Manisa, Türkiye; 20 Department of Infectious Diseases and Clinical Microbiology, Mardin Training and Research Hospital, Mardin, Türkiye; 21 Department of Infectious Diseases and Clinical Microbiology, Kocaeli City Hospital, University of Health Sciences, Kocaeli, Türkiye; 22 Department of Infectious Diseases and Clinical Microbiology, Faculty of Medicine, Istanbul Medipol University, Istanbul, Türkiye; 23 Department of Internal Medicine, Faculty of Medicine, Istanbul Medipol University, Istanbul, Türkiye

**Keywords:** Hepatitis B, Chronic, Fibrosis, Liver, Socioeconomic factor, Area-level deprivation, Health services accessibility, Social determinants of health, Türkiye

## Abstract

**Background:**

Socioeconomic disadvantage may influence the chronic hepatitis B (CHB) care continuum and the stage at which clinically relevant liver disease is recognised. However, evidence linking both individual- and area-level socioeconomic disadvantage to histologically assessed liver fibrosis in CHB remains limited. We examined whether socioeconomic disadvantage at the individual and area levels was associated with liver fibrosis severity in adults with CHB in Türkiye.

**Methods:**

In this nationwide multicentre observational study, adults with CHB who underwent liver biopsy in routine care between 1 January 2019 and 31 December 2024 across 24 centres in Türkiye were screened. Clinical and histopathological data were obtained retrospectively from medical records, while socioeconomic data were collected after biopsy through structured face-to-face interviews. Fibrosis was staged using the Ishak system. Individual socioeconomic indicators and province-level categories of the socioeconomic development index (SEDI) were examined. The primary analysis used ordinal logistic regression. Supportive analyses included multivariable logistic regression, 1:1 propensity score matching with conditional logistic regression, overlap weighting, likelihood-ratio tests for interaction, chi-square or Fisher’s exact tests, Kruskal–Wallis tests, and Wilcoxon rank-sum tests, with Benjamini–Hochberg adjustment for post-hoc comparisons.

**Results:**

The analytic cohort included 956 adults, of whom 53.7% were male. Mild fibrosis (Ishak 0–2) was present in 73.1%, moderate fibrosis (3–4) in 21.7%, and advanced fibrosis/cirrhosis (5–6) in 5.2%. In multivariable ordinal regression, higher fibrosis stage was associated with older age at biopsy (adjusted *OR* = 1.12 per 10-year increase, 95% *CI*: 1.02–1.24; *P* = 0.019), longer travel time to a centre capable of CHB follow-up (adjusted *OR* = 1.53 per category, 95% *CI*: 1.30–1.79; *P* < 0.001), longer duration of residence in the current place of residence (adjusted *OR* = 1.21 per 5-year increase, 95% *CI*: 1.03–1.42; *P* = 0.023), and lower residential socioeconomic development (adjusted *OR* = 1.97 per one-level decrease, 95% *CI*: 1.82–2.15; *P* < 0.001). In matched and overlap-weighted comparisons of SEDI 6 versus SEDI 1, the corresponding estimates were *OR* = 26.00 (95% *CI*: 8.21–82.37; *P* < 0.001) and *OR* = 24.75 (95% *CI*: 8.96–68.36; *P* < 0.001), respectively.

**Conclusions:**

Liver fibrosis severity in adults with CHB was associated more consistently with area-level socioeconomic disadvantage of the current place of residence than with birthplace socioeconomic context or most individual socioeconomic indicators. These findings support a social epidemiological interpretation in which place-based disadvantage may be more closely linked to fibrosis severity at clinical presentation, potentially through unequal access to timely follow-up and specialist assessment.

**Supplementary Information:**

The online version contains supplementary material available at https://doi.org/10.1186/s40249-026-01497-8.

## Background

Despite the availability of an effective vaccine, chronic hepatitis B (CHB) remains a major global cause of cirrhosis and hepatocellular carcinoma. In 2022, an estimated 254 million people were living with hepatitis B virus (HBV) infection, with 1.2 million new infections and 1.1 million deaths, highlighting the continuing gap between preventable infection and persistent disease burden [1, 2]. Within this global context, Türkiye remains a clinically and epidemiologically relevant setting. Available national data suggest that hepatitis B surface antigen (HBsAg) seroprevalence in Türkiye is approximately 4.0%–4.6%, with substantial variation across age groups and regions [3, 4].

Social epidemiology provides a useful framework for understanding how social conditions shape unequal patterns of health across populations [5–7]. Within this framework, individual socioeconomic disadvantage refers to restricted life chances and limited access to the material and social resources that influence everyday life, commonly reflected in education, occupation, income, housing, and related indicators of socioeconomic position [5, 6]. Area-level socioeconomic disadvantage, by contrast, captures the concentration of disadvantage within places and is typically operationalised through multidimensional measures spanning income, employment, education, housing, and access to services [6, 8]. Although conceptually distinct, these dimensions are closely interconnected, because illness is shaped not only by individual circumstances but also by the social and material context in which people live. This perspective is especially relevant to chronic and infectious diseases, in which disadvantage may influence access to care, continuity of follow-up, and subsequent clinical trajectories [5, 7, 8].

Recent HBV research has increasingly linked socioeconomic disadvantage to the pathway from diagnosis to liver-related outcomes. A mixed-method systematic review found that, among marginalised and socioeconomically disadvantaged populations, access to vaccination, screening, treatment, and linkage to care remains consistently constrained, with barriers including low health literacy, lack of insurance, affordability, and travel distance [9]. At the area level, Japanese municipal data showed a positive association between socioeconomic deprivation and HBsAg positivity [10]. In England, primary care cohort data further linked greater deprivation with a higher risk of hepatocellular carcinoma among people living with CHB [11]. Taken together, these findings suggest that social disadvantage may be relevant to HBV not only through reduced access to care, but also through differences in clinically consequential liver outcomes. In this context, fibrosis severity may be viewed as a clinically meaningful correlate of late presentation or delayed care; a consensus definition for chronic viral hepatitis defines late presentation with advanced liver disease as entry into care when substantial liver fibrosis is already present [12].

In Türkiye, area-level socioeconomic context can be characterised using the official “İl SEGE-2025” framework, operationalised here as the province-level socioeconomic development index (SEDI) [13]. This province-level framework is based on 52 indicators across eight domains, including demography, employment, education, health, competitive and innovative capacity, fiscal structure, accessibility, and quality of life, and classifies the 81 provinces into six socioeconomic development levels [13]. As such, it provides a structured measure of spatial inequality beyond single indicators such as income alone. This framework is particularly relevant to chronic hepatitis B, in which place may shape not only access to care, but also the broader social and material conditions through which liver disease is recognised and managed. Against this background, we conducted a national multicentre study in Türkiye integrating face-to-face individual socioeconomic data with SEDI-based area-level classification to examine whether individual and area-level socioeconomic disadvantage were associated with biopsy-based liver fibrosis severity in adults living with CHB, conceptualised as a marker of cumulative liver injury and a clinically meaningful correlate of late presentation or delayed care.

## Methods

### Study design and setting

This nationwide multicentre observational study included adults with CHB who underwent liver biopsy in routine care between 1 January 2019 and 31 December 2024 across 24 centres in Türkiye. Clinical, histopathological, and biopsy-related data were obtained retrospectively from medical records at participating centres, whereas socioeconomic data were collected after biopsy through structured face-to-face interviews; accordingly, interview-based variables reflected reported circumstances at the time of interview rather than necessarily at the time of biopsy. Inclusion in the analytic cohort required available biopsy-based histopathological fibrosis data, outpatient attendance, informed consent, and completion of the structured face-to-face socioeconomic interview. The study was designed and reported in accordance with the Strengthening the Reporting of Observational Studies in Epidemiology (STROBE) statement [16].

### Participants

**Fig. 1:**
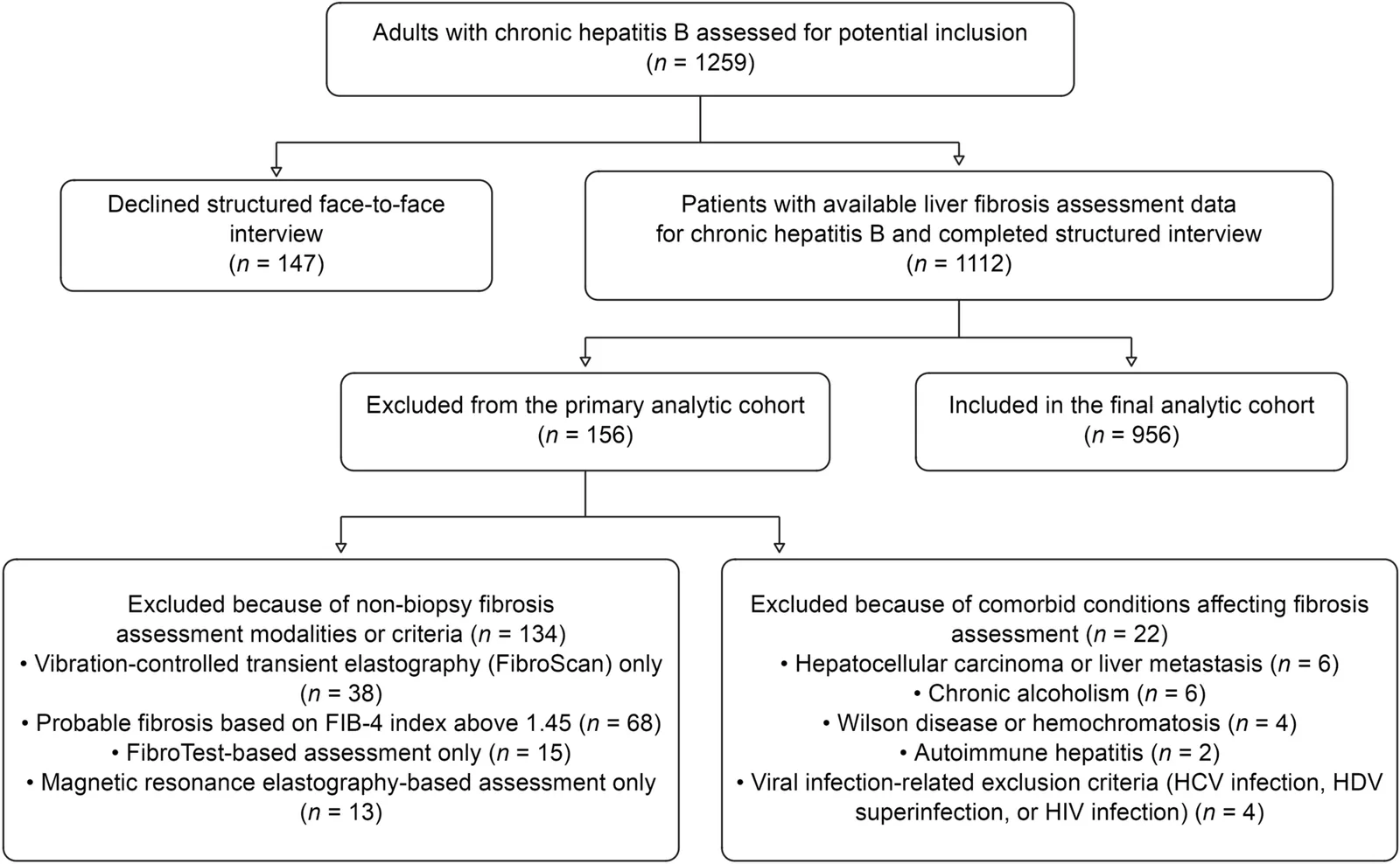
Flowchart of study population. Of 1259 adults with CHB assessed for potential inclusion, 147 declined the structured face-to-face interview. Among the remaining 1112 adults with CHB who had available liver fibrosis assessment data and completed structured face-to-face interview data, 956 were retained in the final analytic cohort after exclusion of those without biopsy-based fibrosis assessment and those with comorbid conditions that could affect fibrosis evaluation. *CHB* chronic hepatitis B; *FIB-4* fibrosis-4 index; *HCV* hepatitis C virus; *HDV* hepatitis D virus; *HIV* human immunodeficiency virus

Eligible participants were adults aged 18 years or older with CHB, defined as persistence of HBsAg for at least 6 months [14], who had undergone liver biopsy and had biopsy material or histopathological data available for review. Patients who declined the structured face-to-face interview were not included in the analytic cohort because interview-administered socioeconomic data could not be obtained. Participants were excluded if they had factors likely to influence liver fibrosis severity independently of CHB, including co-infection with hepatitis C virus, hepatitis D virus, or human immunodeficiency virus, malignancy, chronic alcohol use, or metabolic or genetic liver diseases such as Wilson disease or haemochromatosis (Fig. 1).

### Data collection and study variables

Clinical variables abstracted from medical records included age at HBV diagnosis, age at liver biopsy, sex, documented duration of HBV follow-up before biopsy, follow-up interval, and time from initial recommendation to liver biopsy. Duration of HBV follow-up before biopsy was defined as the documented period of clinical follow-up before liver biopsy rather than the total time elapsed since HBV diagnosis, because some participants had interruptions in care after initial diagnosis. Follow-up interval was defined as the usual interval between routine CHB follow-up visits and was modelled as an ordered variable with three categories (every 3 months, every 6 months, and annually), such that a one-category increase represented a longer interval between visits.

Variables not routinely available in clinical records were obtained through structured face-to-face interviews after biopsy. Interview-administered socioeconomic variables were not abstracted from medical records or imputed when the structured face-to-face interview was not completed. The interval between liver biopsy and the structured face-to-face interview was calculated in months using the recorded biopsy timing and interview period. These included travel time from home to the nearest secondary- or tertiary-care centre capable of providing CHB follow-up, categorised as < 15 min, 15–30 min, 31–60 min, or > 60 min, and time lived in the current place of residence. Accordingly, these measures reflected participants’ reported circumstances at the time of interview rather than necessarily at the time of biopsy.

Socioeconomic variables were grouped into individual-level and area-level indicators. Individual-level indicators included participant and parental education, employment status, household size, monthly household income, per-capita household income, receipt of social assistance, and health insurance or social security coverage. Area-level indicators comprised the socioeconomic development categories assigned to participants’ province of birth and current province of residence according to the official İl SEGE-2025 framework (SEDI) [13]. SEDI classifies the 81 provinces into six socioeconomic development levels; in the present study, SEDI 1 denotes the highest and SEDI 6 the lowest level of socioeconomic development [13]. Accordingly, greater area-level disadvantage corresponded to a lower socioeconomic development level.

### Histopathological assessment and outcome definition

Liver fibrosis was staged histologically using the Ishak system, which ranges from 0 to 6 and reflects increasing fibrosis severity [15]. In the primary analysis, Ishak fibrosis stage was modelled as an ordinal outcome across the full range of categories. For additional analyses, significant fibrosis was defined as Ishak stage ≥ 3.

### Statistical analysis

#### Primary regression analyses

The primary analysis examined associations between deprivation-related indicators and liver fibrosis severity using ordinal logistic regression, with Ishak fibrosis stage (0–6) modelled as an ordered outcome. Effect estimates are presented as odds ratios (*OR*s) with 95% confidence intervals (*CI*s). For interpretability, age at HBV diagnosis and age at biopsy were modelled per 10-year increase, time lived in the current place of residence per 5-year increase, documented duration of HBV follow-up before biopsy per 1-year increase, time from initial recommendation to liver biopsy per 1-month increase, and follow-up interval, travel time, and residential SEDI per one-category change. Candidate variables were examined in univariable analyses and then considered in multivariable ordinal regression models, with model selection informed by the Akaike information criterion and global *P* values for multi-category predictors derived from likelihood-ratio tests. All reported *P* values were two-sided, and *P* < 0.05 was considered statistically significant unless otherwise specified.

#### Additional comparative analyses of SEDI 1 and SEDI 6

Additional comparative analyses focused on participants residing in the two current residential socioeconomic extremes, SEDI 1 and SEDI 6. These analyses included multivariable logistic regression for significant fibrosis adjusted for age at biopsy, sex, and equivalised household income, as well as propensity score matching and overlap weighting. Propensity scores for residence in SEDI 6 versus SEDI 1 were estimated using logistic regression including age at biopsy and sex. These variables were selected because they are temporally antecedent demographic characteristics and are unlikely to be downstream consequences of residential socioeconomic disadvantage. Propensity score matching and overlap weighting were used as supportive demographic-balance analyses rather than definitive causal models. To assess robustness to broader covariate adjustment, sequential logistic regression sensitivity models were fitted for significant fibrosis among participants residing in SEDI 1 or SEDI 6 and are reported as Models 1–3 in Supplementary Table S1. Model 1 adjusted for age at biopsy and sex; Model 2 additionally adjusted for individual socioeconomic variables; and Model 3 additionally included access- and follow-up-related variables, interpreted as exploratory/pathway-adjusted covariates. In separate exploratory sensitivity analyses, individual socioeconomic indicators were entered one at a time into models adjusted for current residential SEDI group, age at biopsy, and sex; these analyses are reported as Models A–D in Supplementary Table S2. Participants were matched 1:1 by nearest-neighbour matching without replacement, with exact matching on sex and a caliper of 0.2 standard deviations of the logit propensity score. Matched analyses were performed using conditional logistic regression stratified by matched pair. Unless otherwise specified as matched or overlap-weighted, regression and interaction analyses were performed in the corresponding unmatched analytic sample. Overlap weights were derived from propensity scores estimated using the same covariates, and covariate balance before and after matching or weighting was assessed using standardised mean differences.

#### Inequality, interaction, prediction, and subgroup analyses

Equivalised household income was calculated by dividing household income by the square root of household size. The relative index of inequality (RII) for equivalised household income was modelled using a ridit-type rank ranging from 0 (highest income position) to 1 (lowest income position). Income was selected for the RII analysis because it provided a rankable individual-level socioeconomic gradient; other individual socioeconomic indicators were not modelled using RII because they were categorical, sparse, or did not represent a continuous hierarchy.

Model-based predicted probabilities of significant fibrosis were estimated from interaction models across the observed ranges of age at biopsy and equivalised household income for SEDI 1 and SEDI 6. Interaction analyses were used to examine whether the association between current residential SEDI and significant fibrosis differed by age, sex, or income level, rather than to directly compare area-level and individual-level socioeconomic effects. *P* values for interaction were obtained using likelihood-ratio tests comparing models with and without the interaction term.

To further evaluate individual socioeconomic indicators, exploratory sensitivity models were fitted among participants residing in SEDI 1 or SEDI 6, with significant fibrosis as the outcome. Each model included current residential SEDI group, age at biopsy, sex, and one individual socioeconomic indicator entered separately; these analyses are reported as Models A–D in Supplementary Table S2.

Subgroup analyses were performed according to birthplace SEDI and current residential destination. Among participants born in SEDI 6, current residential destination was categorised as SEDI 6→6, SEDI 6→5, SEDI 6→4, SEDI 6→3, SEDI 6→2, or SEDI 6→1. Overall differences in significant fibrosis prevalence and fibrosis stage distributions were assessed using the chi-square or Fisher’s exact test, as appropriate, and the Kruskal–Wallis test. Exploratory post-hoc pairwise comparisons used Fisher’s exact or chi-square tests for significant fibrosis and Wilcoxon rank-sum tests for fibrosis stage, with Benjamini–Hochberg adjustment applied only to these post-hoc comparisons.

A reciprocal descriptive subgroup analysis was performed among participants born in SEDI 1 using the same current-residential-destination framework. Because of sparse cell counts, these analyses were treated as descriptive and exploratory.

#### Missing data

Missing data were handled using available-case or model-specific complete-case analysis, depending on the analysis. Descriptive summaries and univariable models used the available non-missing data for each variable or predictor, and denominators therefore varied across variables. For multivariable models, candidate model screening was performed on a common complete-case dataset to allow fair comparison of Akaike information criterion values across candidate models; after model selection, the final model was re-fitted using all participants with complete data for the selected predictors. For propensity score, overlap-weighted, interaction, prediction, and sensitivity analyses, complete-case analysis was applied to the variables included in each corresponding model. No statistical imputation was performed.

All analyses were performed in R version 4.2.2 (R Foundation for Statistical Computing, Vienna, Austria).

## Results

### Study population

A total of 1259 adults with CHB were assessed for potential inclusion, of whom 147 declined the structured face-to-face interview. Among the remaining 1112 adults with available liver fibrosis assessment data and completed interview data, 134 lacked biopsy-based fibrosis assessment and 22 had comorbid conditions that could affect fibrosis evaluation. The final analytic cohort comprised 956 participants with biopsy-based liver fibrosis assessment and completed structured face-to-face interview data (Fig. 1).

### Characteristics of the analytic cohort

Median current age was 46.0 years [37.0–56.0], and 513 participants (53.7%) were male. Most participants were married (*n* = 753, 78.8%), and 612 (64.0%) reported a family history of HBV infection. Median age at HBV diagnosis was 27.0 years [20.0–39.0], whereas median age at biopsy was 41.0 years [32.0–50.0]. The median interval between liver biopsy and the structured face-to-face interview was 60.0 months [24.0–84.0] among participants with available interval data. Mild fibrosis (Ishak 0–2) was present in 73.1% of participants, moderate fibrosis (Ishak 3–4) in 21.7%, and advanced fibrosis/cirrhosis (Ishak 5–6) in 5.2%. Before biopsy, participants had been followed in infectious diseases clinics for CHB for a median of 5.0 years [2.0–10.0], and the median interval from recommendation to biopsy was 1.0 month [1.0–3.0]. Most biopsies were performed in tertiary public hospitals (*n* = 873, 91.3%), and HBV follow-up was conducted at 3-month intervals in 731 participants (76.5%) and at 6-month intervals in 188 (19.7%) (Table 1).

**Table 1:** Demographic, clinical, socioeconomic, healthcare access, and histopathological characteristics of the analytic cohort

Characteristic	Overall cohort (*n* = 956)
Demographic characteristics	
Age, years	46.0 [37.0–56.0]
Sex	
Male	513 (53.7)
Female	441 (46.1)
Other/prefer not to say	2 (0.2)
Marital status	
Single	167 (17.5)
Married	753 (78.8)
Divorced	17 (1.8)
Widowed	19 (2.0)
HBV-related clinical and follow-up characteristics	
Family history of HBV infection	
No	246 (25.7)
Yes	612 (64.0)
Not screened	98 (10.3)
Age at HBV diagnosis, years	27.0 [20.0–39.0]
Duration of HBV follow-up before biopsy, years	5.0 [2.0–10.0]
Age at biopsy, years	41.0 [32.0–50.0]
Biopsy center type	
Tertiary public hospital	873 (91.3)
Public secondary-care hospital	83 (8.7)
Time from initial recommendation to liver biopsy, months	1.0 [1.0–3.0]
Time from liver biopsy to structured face-to-face interview, months	60.0 [24.0–84.0]
Follow-up interval for CHB	
Every 3 months	731 (76.5)
Every 6 months	188 (19.7)
Annually	28 (2.9)
Socioeconomic characteristics	
Birthplace SEDI	
SEDI 1	132 (13.8)
SEDI 2	73 (7.6)
SEDI 3	119 (12.4)
SEDI 4	130 (13.6)
SEDI 5	132 (13.8)
SEDI 6	370 (38.7)
Residential SEDI	
SEDI 1	144 (15.1)
SEDI 2	150 (15.7)
SEDI 3	156 (16.3)
SEDI 4	160 (16.7)
SEDI 5	168 (17.6)
SEDI 6	178 (18.6)
Participant educational level	
Illiterate	52 (5.4)
Literate, no formal education	44 (4.6)
Primary school (5 years)	239 (25.0)
Middle school/8-year compulsory education	120 (12.6)
High school (general/vocational)	251 (26.3)
Associate degree	44 (4.6)
Bachelor’s degree	170 (17.8)
Master’s degree	27 (2.8)
Doctoral degree/PhD	8 (0.8)
Maternal educational level	
Illiterate	402 (42.1)
Literate, no formal education	114 (11.9)
Primary school (5 years)	280 (29.3)
Middle school/8-year compulsory education	82 (8.6)
High school (general/vocational)	34 (3.6)
Bachelor’s degree	9 (0.9)
Paternal educational level	
Illiterate	184 (19.2)
Literate, no formal education	115 (12.0)
Primary school (5 years)	373 (39.0)
Middle school/8-year compulsory education	121 (12.7)
High school (general/vocational)	101 (10.6)
Associate degree	5 (0.5)
Bachelor’s degree	22 (2.3)
Current employment status	
Employed	505 (52.8)
Homemaker	238 (24.9)
Retired	170 (17.8)
Unemployed	36 (3.8)
Student	7 (0.7)
Individual monthly income, TRY per month	28,000.0 [5250.0–60,000.0]
Total monthly household income, TRY per month	50,000.0 [28,000.0–81,250.0]
Household size	4.0 [2.0–5.0]
Receipt of social assistance	
No	898 (93.9)
Yes	58 (6.1)
Household and social environment	
Raised by both parents	
Yes	830 (86.8)
No	126 (13.2)
Housing tenure status	
Owner-occupied housing	630 (66.0)
Rented housing	251 (26.3)
Living with family/relatives	68 (7.1)
Earthquake-related temporary container housing	3 (0.3)
Institution-provided housing	3 (0.3)
Health insurance/social security coverage	
No health insurance/social security coverage	8 (0.8)
Income-tested public health coverage (state-paid premiums; former Green Card)	54 (5.6)
General Health Insurance	874 (91.4)
Private health insurance	3 (0.3)
General Health Insurance plus private health insurance	17 (1.8)
Adverse home environment conditions	
Dampness/mold	92 (9.6)
Inadequate heating	126 (13.2)
Overcrowding	61 (6.4)
Noise	80 (8.4)
Healthcare access characteristics	
Travel time from home to a centre capable of CHB follow-up	418 (43.7)
< 15 min	432 (45.2)
15–30 min	53 (5.5)
31–60 min	53 (5.5)
Ability to access needed healthcare services in the past 12 months	
Always able to access	773 (80.9)
Sometimes unable to access	174 (18.2)
Usually unable to access	9 (0.9)
Histopathologic characteristics	
Fibrosis stage (Ishak)	
Mild fibrosis (0–2)	699 (73.1)
Moderate fibrosis (3–4)	207 (21.7)
Advanced fibrosis/cirrhosis (5–6)	50 (5.2)
Necroinflammatory activity score (modified Knodell/Ishak)	6.0 [5.0–8.0]

Data are presented as median [Q1–Q3] for continuous variables and *n* (%) for categorical variables. Percentages were calculated using non-missing data for each variable. Multiple responses were possible for home environment items; therefore, percentages do not sum to 100%. Income variables were recorded in nominal Turkish lira (TRY) at the time of interview. SEDI 1 indicates the highest and SEDI 6 the lowest socioeconomic development level according to the official “İl SEGE-2025” framework [13]. Follow-up interval data were available for 947 participants. The biopsy-to-interview interval was available for 947 participants. *CHB* chronic hepatitis B; *HBV* hepatitis B virus; *Q1* first quartile; *Q3* third quartile; *SEDI* socioeconomic development index category; *TRY* Turkish lira

### Univariable ordinal regression analysis in the full unmatched cohort

In univariable ordinal regression analyses, male sex was associated with higher odds of a higher fibrosis stage (*OR* = 1.38, 95% *CI:* 1.10–1.74;* P* = 0.006). Both older age at HBV diagnosis (*OR* = 1.10 per 10-year increase, 95% *CI*: 1.02–1.18; *P* = 0.010) and older age at biopsy (*OR* = 1.17 per 10-year increase, 95% *CI*: 1.07–1.28; *P* < 0.001) were likewise associated with higher fibrosis stage. By contrast, a longer follow-up interval for CHB was associated with lower odds of a higher fibrosis stage (*OR* = 0.42 per one-category longer interval, 95% *CI*: 0.33–0.54; *P* < 0.001). Among socioeconomic variables, time lived in the current place of residence showed a modest positive association (*OR* = 1.17 per 5-year increase, 95% *CI*: 1.00–1.36; *P* = 0.046), whereas residential SEDI demonstrated a strong gradient, with lower socioeconomic development associated with markedly higher odds of a higher fibrosis stage (*OR* = 2.06 per one-level lower SEDI group, 95% *CI*: 1.90–2.24; *P* < 0.001). Longer travel time to a centre capable of CHB follow-up was also associated with higher fibrosis stage (*OR* = 1.65 per one-category longer travel time, 95% *CI*: 1.42–1.92; *P* < 0.001) (Table 2).

**Table 2:** Univariable ordinal logistic regression associations with higher Ishak fibrosis stage in the full unmatched analytic cohort

Characteristic	Category/unit	*n* available	*OR* for higher fibrosis stage	95% *CI*	*P* value
Demographic characteristics					
Sex^a^	Male vs Female	954	**1.38**	**1.10–1.74**	**0.006**
Marital status^a^	Married vs Single	920	1.18	0.88–1.60	0.271
HBV-related clinical and follow-up characteristics					
Family history of HBV infection among participants with a positive family history only^b^		612			0.185
	First-degree relative affected		Reference		
	Other relative affected		0.72	0.51–1.02	0.067
	Multiple relatives affected		0.83	0.57–1.23	0.357
Age at HBV diagnosis, years^a^	Per 10-year increase	955	**1.10**	**1.02–1.18**	**0.010**
Duration of HBV follow-up before liver biopsy, years^a^	Per 1-year increase	956	0.98	0.96–1.00	0.056
Age at biopsy, years^a^	Per 10-year increase	956	**1.17**	**1.07–1.28**	**< 0.001**
Time from initial recommendation to liver biopsy, months^a^	Per 1-month increase	935	1.00	1.00–1.01	0.379
Follow-up interval for CHB^a^	Per one-category longer interval (Every 3 months → Every 6 months → Annually)	947	**0.42**	**0.33–0.54**	**< 0.001**
Socioeconomic characteristics					
ime lived in the current place of residence^a^	Per 5-year increase	956	**1.17**	**1.00–1.36**	**0.046**
Birthplace SEDI^a^	Per one-level lower SEDI group (SEDI 1 → SEDI 6)	956	1.01	0.95–1.08	0.747
Residential SEDI^a^	Per one-level lower SEDI group (SEDI 1 → SEDI 6)	956	**2.06**	**1.90–2.24**	**< 0.001**
Current employment status^b^		956			0.063
	Employed		Reference		
	Homemaker		0.87	0.66–1.14	0.307
	Retired		**1.45**	**1.04–2.01**	**0.027**
	Student		0.71	0.21–2.37	0.577
	Unemployed		0.77	0.42–1.42	0.400
Participant educational level^a^	Per one-category higher educational level*	955	0.99	0.93–1.05	0.648
Maternal educational level^a^	Per one-category higher educational level*	921	1.04	0.95–1.14	0.411
Paternal educational level^a^	Per one-category higher educational level*	921	1.00	0.92–1.08	0.965
Per-capita household monthly income, 1000 TRY/month^a^	Per 1000 TRY/month increase	893	1.00	1.00–1.01	0.901
Receipt of social assistance^a^	Yes vs No	956	0.62	0.39–1.00	0.051
Household and social environment					
Health insurance/social security coverage^b^		956			0.522
	General Health Insurance		Reference		
	General Health Insurance + private insurance		1.32	0.58–2.99	0.503
	Other (No health insurance/social security coverage)^c^		0.52	0.14–1.87	0.315
	Private insurance only		0.36	0.06–2.22	0.269
	State-paid public coverage		1.21	0.72–2.01	0.473
Raised by both parents^a^	Yes vs No	956	0.89	0.63–1.25	0.500
Housing tenure status^a^		955			0.461
	Owner-occupied		Reference		
	Living with family/relatives		0.82	0.53–1.28	0.376
	Other (Earthquake-related temporary container housing; Institution-provided housing)^c^		1.40	0.36–5.35	0.627
	Rented		0.83	0.64–1.09	0.178
Adverse home environment conditions					
Dampness/mold^a^	Yes vs No	956	0.76	0.52–1.13	0.178
Inadequate heating^a^	Yes vs No	956	0.85	0.60–1.19	0.349
Overcrowding^a^	Yes vs No	956	1.35	0.86–2.13	0.187
Noise^a^	Yes vs No	956	1.45	0.96–2.20	0.079
Healthcare access characteristics					
Travel time from home to a centre capable of CHB follow-up^a^	Per one-category longer travel time (< 15 → 15–30 → 31–60 → > 60 min)	956	**1.65**	**1.42–1.92**	**< 0.001**
Ability to access needed healthcare services in the past 12 months^b^		956			0.315
	Always able to access		Reference		
	Sometimes unable to access		1.26	0.94–1.69	0.129
	Usually unable to access		1.05	0.33–3.38	0.933

Odds ratios (*OR*s) represent the odds of being in a higher fibrosis stage category

^a^ *OR*s, 95% confidence intervals (*CI*s), and *P* values were obtained from univariable ordinal logistic regression models. ^b^ For multi-category predictors, the *P* value shown on the variable row is the global *P* value from the univariable ordinal logistic regression model; category-specific *OR*s and 95% *CI*s are shown relative to the reference category. ^c^ Rare categories were collapsed where necessary and are shown explicitly in parentheses

^*^ Educational level variables (participant, mother, and father) were modelled as ordinal variables in the following order: Illiterate, Literate with no formal education, Primary school (5 years), Middle school/8-year compulsory education, High school (general/vocational), Associate degree, Bachelor's degree, Master's degree, and Doctoral degree/PhD

For marital status, analyses were restricted to married and single participants. For family history, analyses were restricted to participants with a positive family history of hepatitis B infection, and the reference category was first-degree relative affected. SEDI 1 indicates the highest and SEDI 6 the lowest socioeconomic development level according to the Official “İl SEGE-2025” framework [13]. Available-case a*n*alysis was used for each u*n*ivariable model; therefore, *n* available may differ across predictors. Statistically significant results (*P* < 0.05) are shown in bold. Continuous predictors were analysed as follows: Age at biopsy and age at HBV diagnosis per 10-year increase; time lived in the current place of residence per 5-year increase; duration of HBV follow-up before liver biopsy per 1-year increase; time from initial recommendation to liver biopsy per 1-month increase; per-capita household monthly income per 1000 TRY per month increase

*HBV* hepatitis B virus; *OR* odds ratio; *CI* confidence interval; *SEDI* socioeconomic development index category; *TRY* Turkish lira

### Multivariable ordinal regression analysis in the full unmatched cohort

In multivariable ordinal logistic regression analysis, older age at biopsy (adjusted *OR* = 1.12 per 10-year increase, 95% *CI*: 1.02–1.24; *P* = 0.019), longer travel time to a centre capable of CHB follow-up (adjusted *OR* = 1.53 per one-category longer travel time, 95% *CI*: 1.30–1.79; *P* < 0.001), shorter documented duration of HBV follow-up before biopsy (adjusted *OR* = 0.98 per 1-year increase, 95% *CI*: 0.96–1.00; *P* = 0.035), greater time lived in the current place of residence (adjusted *OR* = 1.21 per 5-year increase, 95% *CI*: 1.03–1.42; *P* = 0.023), and lower residential SEDI (adjusted *OR* = 1.97 per one-level decrease in socioeconomic development, 95% *CI*: 1.82–2.15; *P* < 0.001) were independently associated with higher fibrosis stage, whereas a longer follow-up interval for CHB was associated with lower odds of more advanced fibrosis (adjusted *OR* = 0.49 per one-category longer interval, 95% *CI*: 0.38–0.63; *P* < 0.001). Receipt of social assistance was likewise associated with lower odds of a higher fibrosis stage (adjusted *OR* = 0.53, 95% *CI*: 0.32–0.86; *P* = 0.011) (Table 3).

**Table 3:** Multivariable ordinal logistic regression associations with higher Ishak fibrosis stage in the full unmatched analytic cohort

Characteristic	Category/unit	Adjusted *OR* (95% *CI*)	*P* value
Sex	Male vs female	1.24 (0.97–1.58)	0.081
Age at biopsy, years	Per 10-year increase	1.12 (1.02–1.24)	0.019
Follow-up interval for CHB	Every 3 months/every 6 months/annually; per one-category longer interval	0.49 (0.38–0.63)	< 0.001
Travel time from home to a centre capable of CHB follow-up	< 15 min/15–30 min/31–60 min/> 60 min; per one–category longer travel time	1.53 (1.30–1.79)	< 0.001
Duration of HBV follow-up before biopsy, years	Per 1-year increase	0.98 (0.96–1.00)	0.035
Time lived in the current place of residence	Per 5-year increase	1.21 (1.03–1.42)	0.023
Residential SEDI	Per one-level decrease in socioeconomic development (SEDI 1 to SEDI 6)	1.97 (1.82–2.15)	< 0.001
Receipt of social assistance	Yes vs No	0.53 (0.32–0.86)	0.011

Adjusted odds ratios (*ORs*) represent the odds of being in a higher fibrosis stage category in the multivariable ordinal logistic regression model. Continuous predictors were modelled per unit shown in the Category/unit column. *OR* odds ratio; C*H*B chronic hepatitis B; *HBV* hepatitis B virus; *CI* confidence interval; *SEDI* socioeconomic development index category

### Matched comparison of extreme residential SEDI groups

**Fig. 2:**
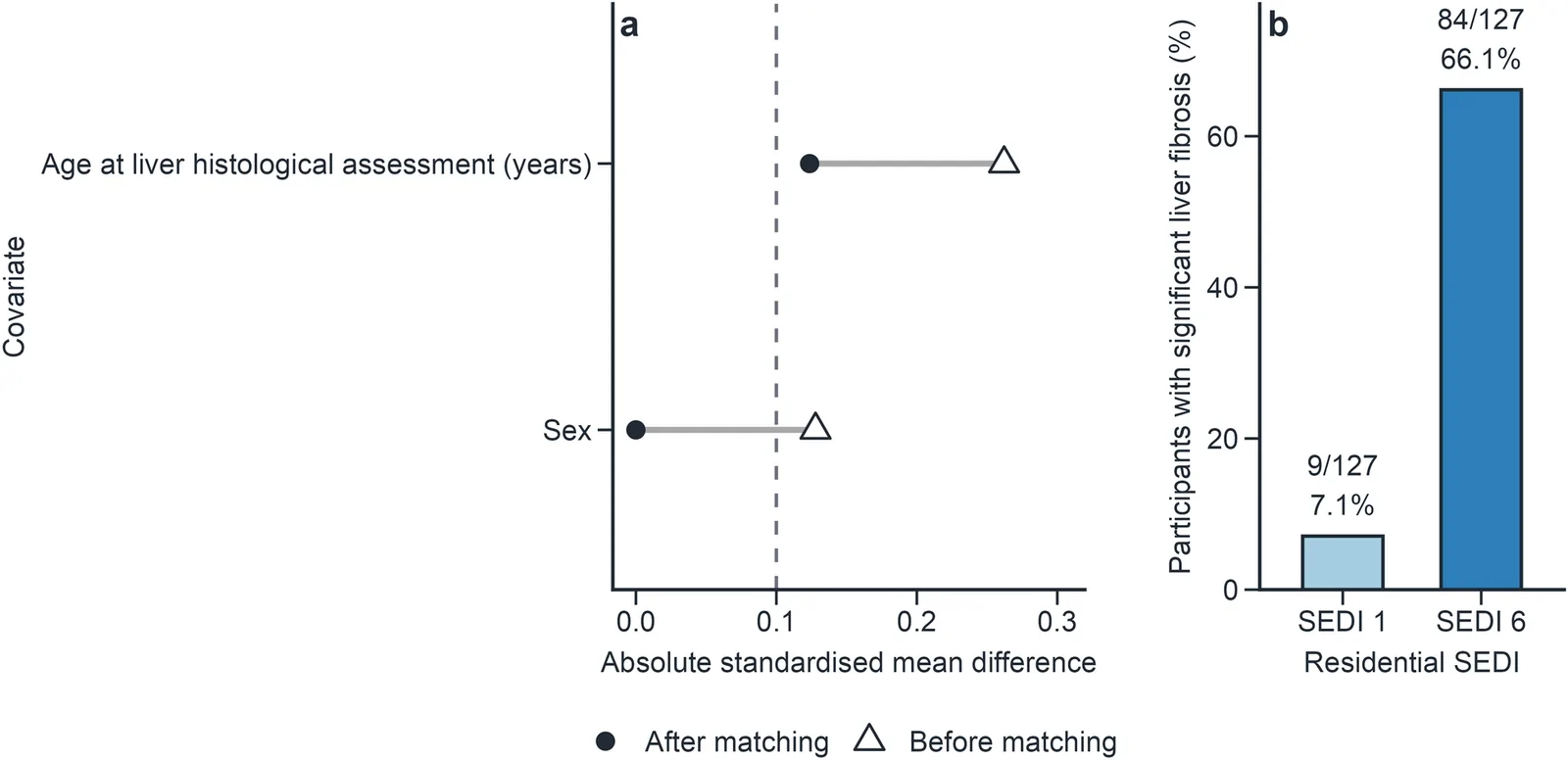
Covariate balance and matched fibrosis risk in SEDI 1 and SEDI 6. **a** Absolute standardised mean differences before and after 1:1 caliper propensity score matching. **b** The prevalence of significant liver fibrosis (Ishak stage ≥ 3) in the matched cohort. A total of 127 matched pairs were retained. In the matched analysis, residence in SEDI 6 was associated with higher odds of significant liver fibrosis than residence in SEDI 1 (*OR* = 26.00, 95% *CI*: 8.21–82.37; *P* < 0.001). *SEDI* socioeconomic development index category; *CI* confidence interval; *OR* odds ratio

In a supportive propensity score-matched analysis restricted to participants residing in the two current residential socioeconomic extremes, SEDI 1 and SEDI 6, 1:1 caliper matching was performed using age at biopsy and sex, with exact matching on sex. This yielded 127 matched pairs. After matching, covariate balance between the two groups improved substantially, whereas differences in fibrosis severity remained pronounced. In the matched sample, median fibrosis stage was 1.0 [0.0–2.0] in SEDI 1 and 3.0 [2.0–4.0] in SEDI 6; significant liver fibrosis (Ishak ≥ 3) was present in 9 participants (7.1%) in SEDI 1 and 84 (66.1%) in SEDI 6. In conditional logistic regression stratified by matched pair, residence in SEDI 6 was associated with higher odds of significant liver fibrosis than residence in SEDI 1 (*OR* = 26.00, 95% *CI*: 8.21–82.37; *P* < 0.001) (Table 4, Fig. 2).

**Table 4:** Covariate balance and fibrosis outcomes before and after 1:1 propensity score matching of residential SEDI 1 and SEDI 6 groups

Characteristic	Before matching SEDI 1 (*n* = 144)	Before matching SEDI 6 (*n* = 178)	After matching SEDI 1 (*n* = 127)	After matching SEDI 6 (*n* = 127)
Sample size, *n*	144	178	127	127
Age at biopsy, years	40.1 ± 10.9	43.7 ± 13.6	40.7 ± 11.0	42.4 ± 11.1
Sex,* n* (%)				
Female	71 (49.3%)	65 (36.5%)	54 (42.5%)	54 (42.5%)
Male	73 (50.7%)	111 (62.4%)	73 (57.5%)	73 (57.5%)
Other/prefer not to say	0 (0.0%)	2 (1.1%)	0 (0.0%)	0 (0.0%)
Fibrosis stage, median [Q1–Q3]	1.0 [0.0–2.0]	3.0 [2.0–4.0]	1.0 [0.0–2.0]	3.0 [2.0–4.0]
Significant liver fibrosis (Ishak ≥ 3), *n* (%)	9 (6.2%)	119 (66.9%)	9 (7.1%)	84 (66.1%)

Matching was performed using 1:1 nearest-neighbour propensity score matching without replacement, based on age at biopsy and sex, with exact matching on sex and a caliper of 0.2 standard deviations of the logit propensity score

*SEDI* socioeconomic development index category; *CI* confidence interval; *OR* odds ratio. In conditional logistic regression stratified by matched pair, residence in SEDI 6 versus SEDI 1 was associated with significant liver fibrosis (*OR* = 26.00, 95% *CI*: 8.21–82.37; *P* < 0.001)

### Overlap-weighted comparison of socioeconomic extremes

**Fig. 3:**
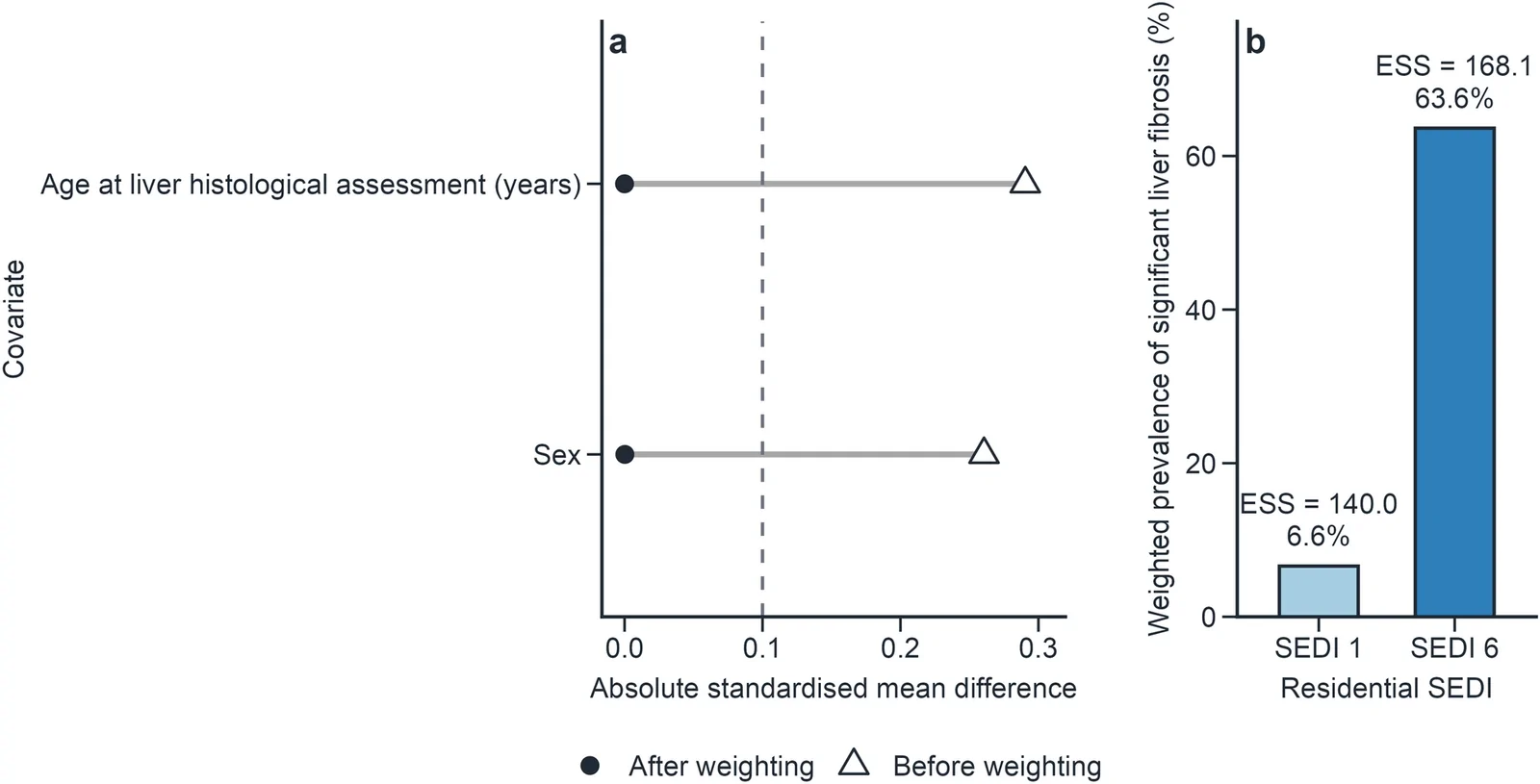
Covariate balance and weighted fibrosis risk after overlap weighting. **a** Absolute standardised mean differences before and after overlap weighting. **b** The weighted prevalence of significant liver fibrosis (Ishak stage ≥ 3) in SEDI 1 and SEDI 6, with effective sample size shown for each group. In the weighted analysis, residence in SEDI 6 was associated with higher odds of significant liver fibrosis than residence in SEDI 1 (*OR* = 24.75, 95% *CI*: 8.96–68.36; *P* < 0.001). *SEDI* socioeconomic development index category; *ESS* effective sample size; *CI* confidence interval; *OR* odds ratio

In a supportive overlap-weighted analysis restricted to participants residing in the two current residential socioeconomic extremes, SEDI 1 and SEDI 6, weighting improved balance in age at biopsy and sex between groups. After weighting, the weighted mean age was 41.4 ± 10.9 years in SEDI 1 and 41.4 ± 13.2 years in SEDI 6, and the sex distribution was closely aligned. Differences in fibrosis burden nevertheless remained substantial: the weighted prevalence of significant liver fibrosis (Ishak ≥ 3) was 6.6% in SEDI 1 and 63.6% in SEDI 6. In the weighted outcome model, residence in SEDI 6 was associated with higher odds of significant liver fibrosis than residence in SEDI 1 (*OR* = 24.75, 95% *CI*: 8.96–68.36; *P* < 0.001) (Table 5, Fig. 3).

**Table 5:** Covariate balance, effective sample sizes, and fibrosis outcomes before and after overlap weighting of residential SEDI 1 and SEDI 6 groups

Characteristic	Before weighting: SEDI 1 (*n* = 144)	Before weighting: SEDI 6 (*n* = 178)	After weighting: SEDI 1 (ESS = 140.0)	After weighting: SEDI 6 (ESS = 168.1)
Observed sample size/effective sample size	144	178	140.0	168.1
Age at biopsy, years	40.1 ± 10.9	43.7 ± 13.6	41.4 ± 10.9	41.4 ± 13.2
Sex, %				
Female	71 (49.3%)	65 (36.5%)	43.2%	43.2%
Male	73 (50.7%)	111 (62.4%)	56.8%	56.8%
Other/prefer not to say	0 (0.0%)	2 (1.1%)	0.0%	0.0%
Significant liver fibrosis (Ishak ≥ 3), %	9 (6.2%)	119 (66.9%)	6.6%	63.6%

Overlap weights were derived from propensity scores estimated using age at biopsy and sex. For SEDI 6, weights were defined as 1 minus the propensity score; for SEDI 1, weights were defined as the propensity score. After-weighting columns are presented using weighted summaries, and effective sample size (ESS) is shown for the weighted groups. Significant liver fibrosis was defined as Ishak fibrosis stage ≥ 3. In the overlap-weighted logistic regression model, residence in SEDI 6 versus SEDI 1 was associated with significant liver fibrosis (*OR* = 24.75, 95% *CI*: 8.96–68.36; *P* < 0.001). *SEDI* socioeconomic development index category; *ESS* effective sample size; *CI* confidence interval; *OR* odds ratio

To address potential confounding beyond age and sex, a regression-based sensitivity analysis was performed among participants residing in SEDI 1 or SEDI 6. Current residence in SEDI 6 remained associated with significant liver fibrosis after adjustment for age at biopsy and sex (*OR* = 28.83, 95% *CI*: 13.50–61.57), after additional adjustment for individual socioeconomic variables (*OR* = 28.17, 95% *CI*: 12.65–62.69), and after exploratory adjustment for access- and follow-up-related variables (*OR* = 18.98, 95% *CI*: 8.43–42.72; all *P* < 0.001; Supplementary Table S1).

### Interaction analyses, income-related RII, and individual socioeconomic status sensitivity analyses

**Fig. 4:**
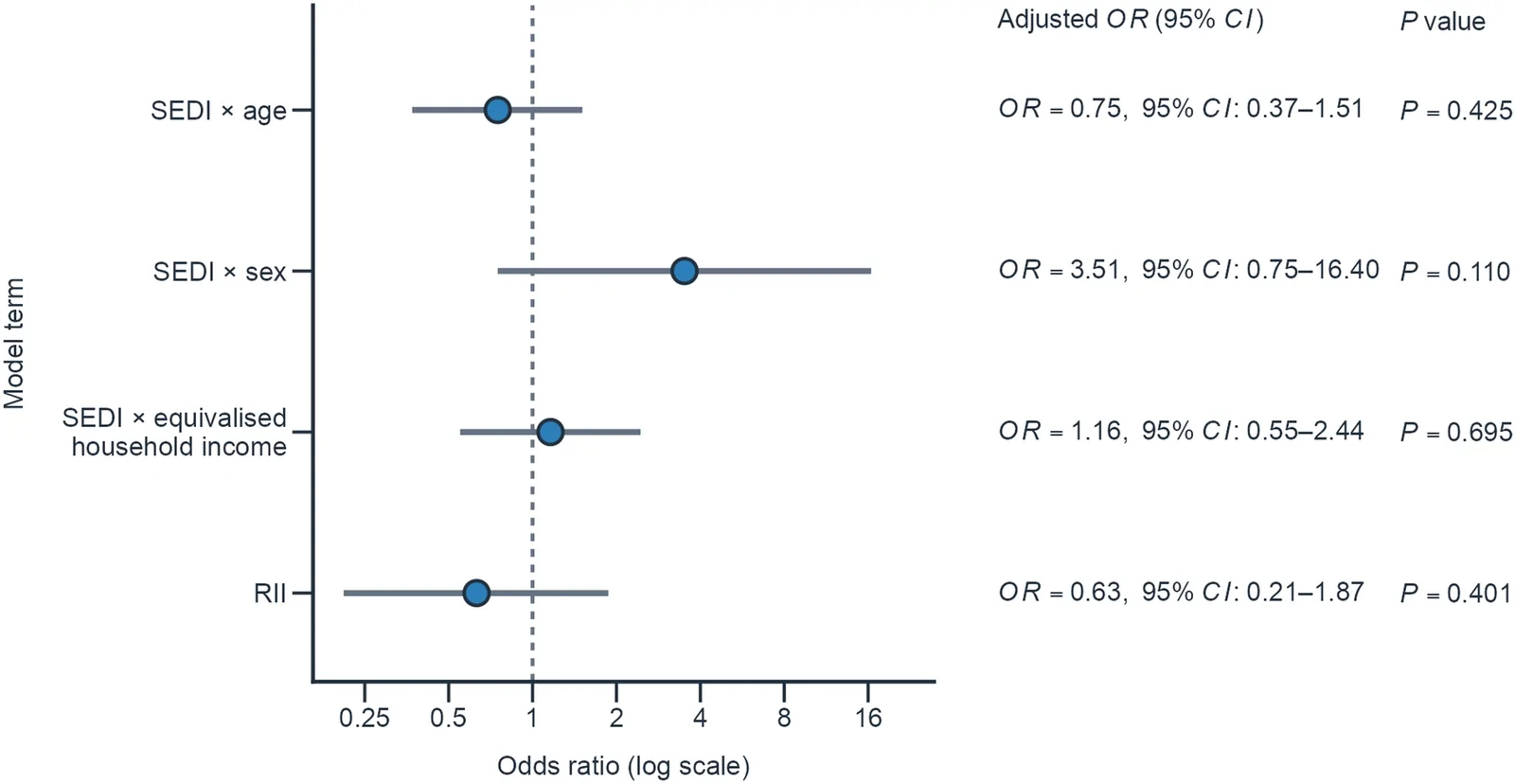
Interaction effects and income-related inequality in significant liver fibrosis. Adjusted odds ratios with 95% confidence intervals are shown for the interactions between the current residential socioeconomic development index (SEDI) and age at liver histological assessment, sex, and equivalised household income, and for the relative index of inequality (RII). Interaction *P* values were obtained from likelihood-ratio tests comparing models with and without each interaction term; the RII *P* value was obtained from the Wald test for the ridit-based income-rank coefficient. The dashed vertical line indicates the null value (odds ratio = 1.0). *SEDI* socioeconomic development index category; RII relative index of inequality; *OR* odds ratio; *CI* confidence interval

In unmatched regression analyses restricted to participants residing in SEDI 1 or SEDI 6 and adjusted for age at biopsy, sex, and equivalised household income, residence in SEDI 6 remained strongly associated with significant liver fibrosis compared with residence in SEDI 1 (*OR* = 24.89, 95% *CI*: 11.54–53.68; *P* < 0.001). Interaction analyses were used to assess whether this association differed across age, sex, or income levels. There was no evidence of effect modification by age (*P* for interaction = 0.425), sex (*P* for interaction = 0.110), or equivalised household income (*P* for interaction = 0.695). In the same restricted analytic sample, the income-related RII was not associated with significant liver fibrosis after adjustment for age at biopsy, sex, and SEDI (*OR* = 0.63, 95% *CI*: 0.21–1.87; *P* = 0.401) (Table 6, Fig. 4).

**Table 6:** Interaction analyses and income-related inequality for significant liver fibrosis among participants residing in SEDI 1 or SEDI 6

Analysis	Interpretation	Adjusted *OR* (95% *CI*)	*P* value/*P* for interaction
Main SEDI effect	SEDI 6 vs SEDI 1, adjusted for age, sex, and equivalised income	24.89 (11.54–53.68)	< 0.001
SEDI × age	Interaction term; ratio of SEDI odds ratios per 10-year increase in age at biopsy	0.75 (0.37–1.51)	0.425
SEDI × sex	Interaction term; ratio of SEDI odds ratios in males vs females	3.51 (0.75–16.40)	0.110
SEDI × equivalised household income	Interaction term; ratio of SEDI odds ratios per 1 SD increase in log equivalised household income	1.16 (0.55–2.44)	0.695
RII	Relative index of inequality for equivalised household income (lowest vs highest income rank), adjusted for age at biopsy, sex, and SEDI	0.63 (0.21–1.87)	0.401

Significant liver fibrosis was defined as Ishak fibrosis stage ≥ 3. The main model was adjusted for age at biopsy, sex, and equivalised household income. Interaction models were fitted separately for SEDI × age at biopsy, SEDI × sex, and SEDI × equivalised household income. Equivalised income was defined as household income divided by the square root of household size. Relative index of inequality (RII) for equivalised household income was modelled using a ridit-type income rank ranging from 0 (highest income position) to 1 (lowest income position). For interaction analyses, the *OR*s represent interaction-term estimates, and *P* values are likelihood-ratio-test *P* values comparing models with and without the interaction term. For the main SEDI effect and RII estimate, *P* values are Wald *P* values from the corresponding regression models. Abbreviations: SEDI socioeconomic development index category; RII relative index of inequality; *OR* odds ratio; *CI* confidence interval; SD standard deviation

In exploratory sensitivity models separately incorporating individual socioeconomic indicators, the association between residence in SEDI 6 and significant fibrosis remained strong after adjustment for equivalised household income, participant educational level, receipt of social assistance, and health insurance/social security coverage (Supplementary Table S2). The adjusted *OR* for SEDI 6 versus SEDI 1 ranged from 24.89 to 29.97 across these models, with all *P* values < 0.001. Equivalised household income, participant educational level, and health insurance/social security coverage were not independently associated with significant fibrosis in these exploratory models, whereas receipt of social assistance showed an inverse association (*OR* = 0.25, 95% *CI*: 0.07–0.97; *P* = 0.046).

### Model-based predicted probabilities of significant liver fibrosis

**Fig. 5:**
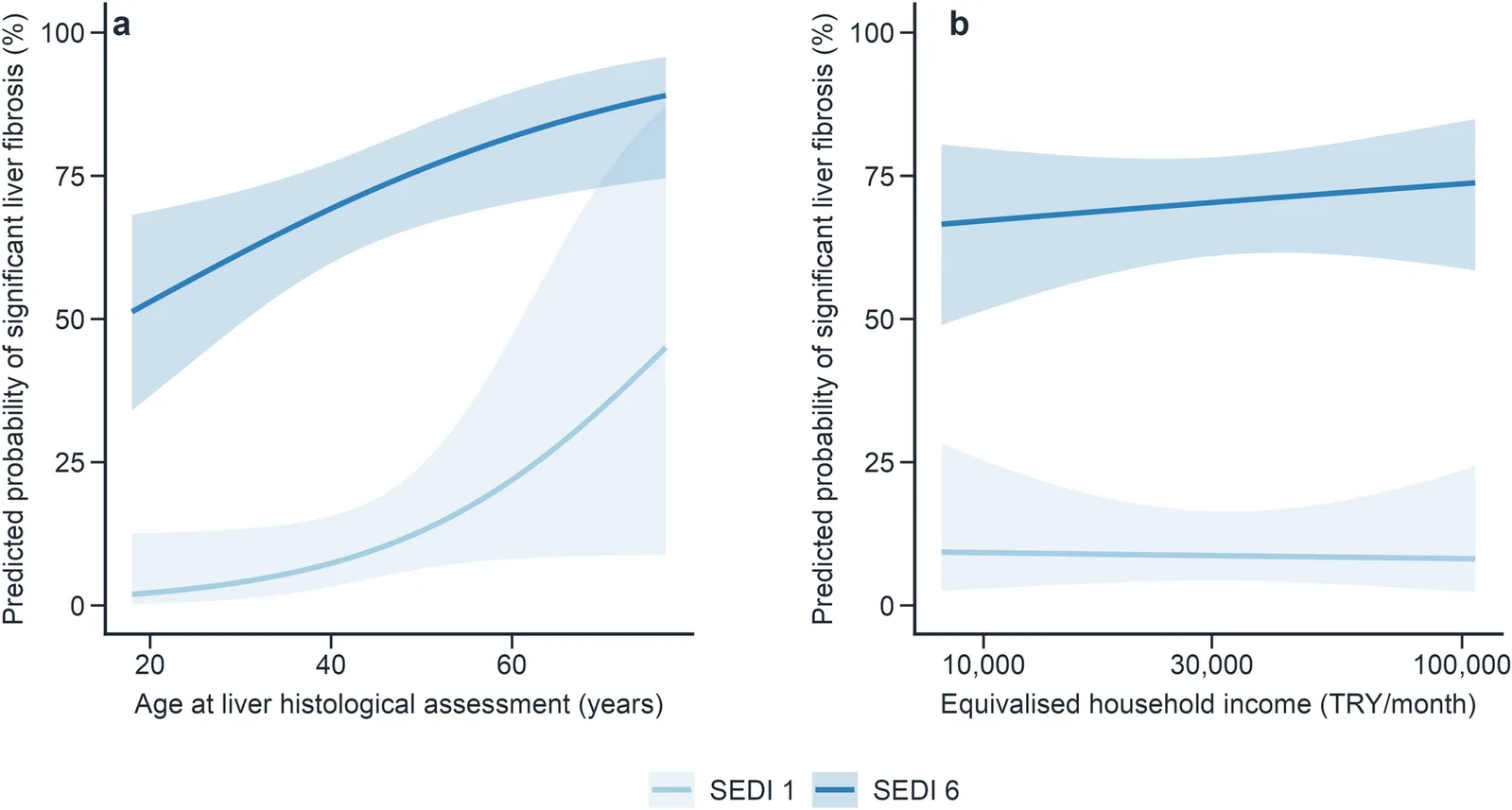
Model-based probabilities of significant liver fibrosis by age and equivalised household income. **a** The predicted probability of significant liver fibrosis according to age at biopsy in SEDI 1 and SEDI 6. **b** The predicted probability according to equivalised household income in SEDI 1 and SEDI 6. Shaded bands indicate 95% confidence intervals. Significant liver fibrosis was defined as Ishak stage ≥ 3. Equivalised household income was defined as household income divided by the square root of household size. *SEDI* socioeconomic development index category; *CI* confidence interval; *OR* odds ratio

Model-based predicted probabilities were consistently higher in SEDI 6 than in SEDI 1. At age 40 years, with sex set to male and log equivalised household income held at its mean, the predicted probabilities were 69.3% (95% *CI*: 59.8–77.4%) in SEDI 6 and 7.4% (95% *CI*: 3.3–15.6%) in SEDI 1. At an equivalised household income of TRY 30,000 per month, with age fixed at the sample median of 41 years and sex set to male, the corresponding probabilities were 70.3% (95% *CI*: 61.0–78.2%) and 8.7% (95% *CI*: 4.4–16.4%), respectively. Predicted probabilities increased with age in both groups, whereas the trajectories across equivalised household income levels were broadly parallel (Fig. 5).

### Birthplace SEDI 6 subgroup: current residential destination and significant fibrosis

**Fig. 6:**
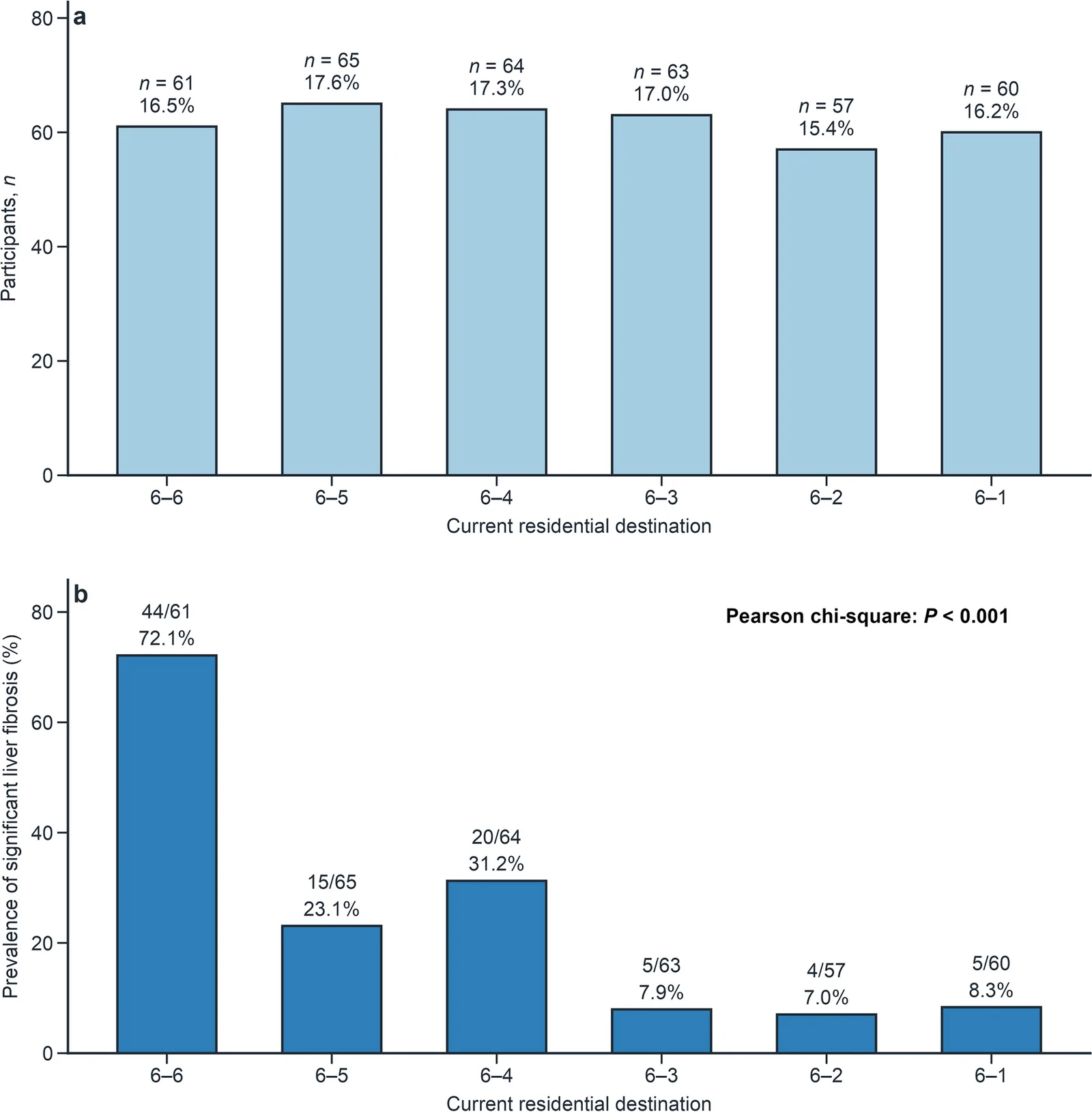
Migration destination and significant fibrosis among participants born in SEDI 6. **a** The distribution of current residential destination groups among participants whose birthplace was classified as SEDI 6. **b** The prevalence of significant fibrosis (Ishak stage ≥ 3) across the same destination groups. The highest prevalence of significant fibrosis was observed among participants who remained in SEDI 6. Overall differences across groups were significant (Pearson chi-square *P* < 0.001). *SEDI* socioeconomic development index category

Among participants whose birthplace was classified as SEDI 6, current residential destination groups were broadly evenly distributed: 61 participants (16.5%) remained in SEDI 6, 65 (17.6%) resided in SEDI 5, 64 (17.3%) in SEDI 4, 63 (17.0%) in SEDI 3, 57 (15.4%) in SEDI 2, and 60 (16.2%) in SEDI 1. However, the prevalence of significant fibrosis differed markedly across destination groups. It was highest among participants who remained in SEDI 6 (44/61, 72.1%), followed by those residing in SEDI 4 (20/64, 31.2%) and SEDI 5 (15/65, 23.1%), and was substantially lower among those residing in SEDI 3 (5/63, 7.9%), SEDI 2 (4/57, 7.0%), and SEDI 1 (5/60, 8.3%). Overall differences across destination groups were significant (Pearson chi-square *P* < 0.001) (Fig. 6). Post-hoc pairwise comparisons, when examined, were interpreted using Benjamini–Hochberg-adjusted *P* values.

### Birthplace SEDI 1 subgroup: current residential destination and significant fibrosis

**Fig. 7:**
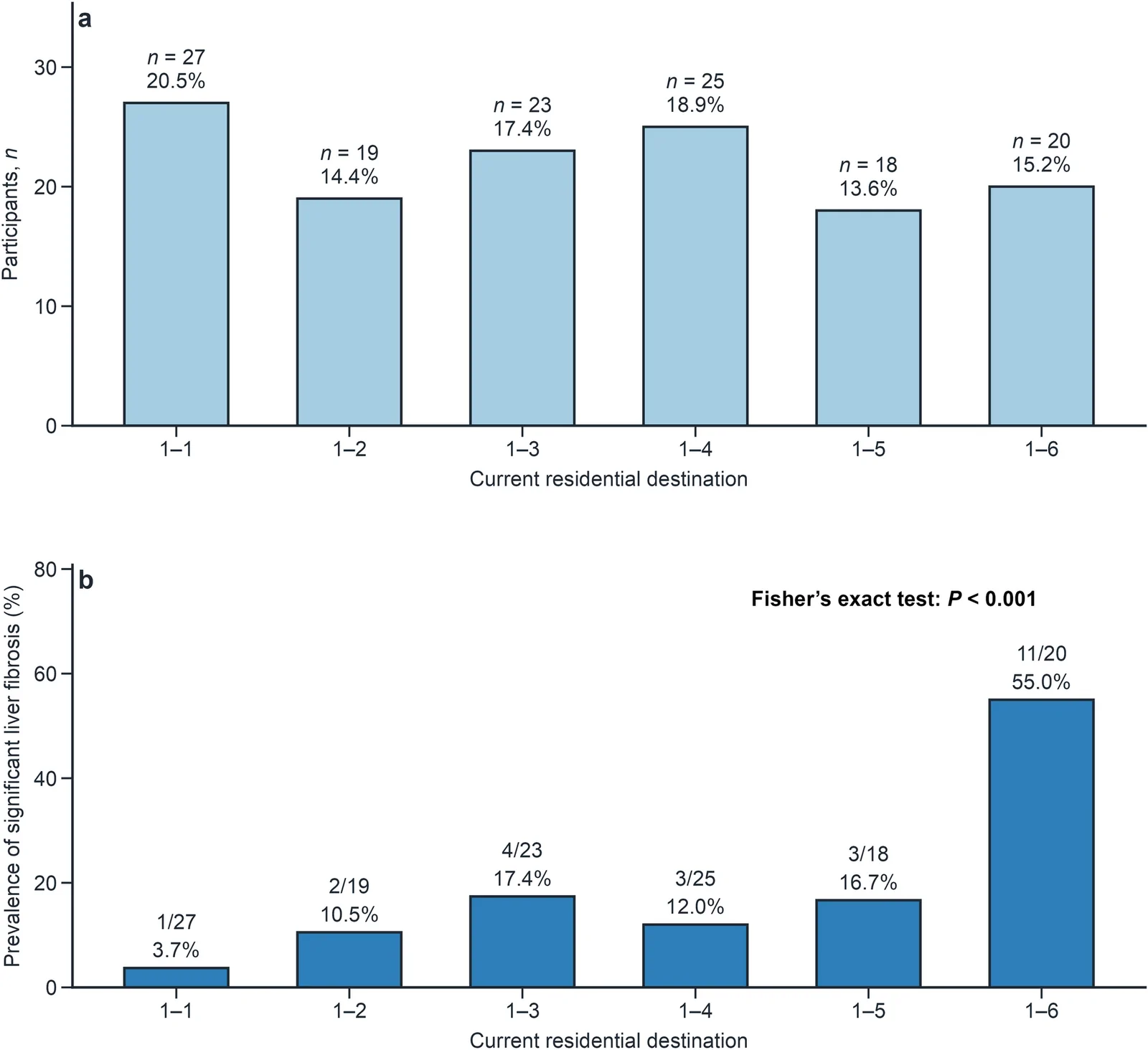
Migration destination and significant fibrosis among participants born in SEDI 1. **a** The distribution of current residential destination groups among participants whose birthplace was classified as SEDI 1. **b** The prevalence of significant fibrosis (Ishak stage ≥ 3) across the same destination groups. The highest prevalence of significant fibrosis was observed among participants residing in SEDI 6. Overall differences across groups were significant (Fisher’s exact *P* < 0.001). *SEDI* socioeconomic development index category

Among participants whose birthplace was classified as SEDI 1, current residential destination groups were distributed as follows: 27 participants (20.5%) remained in SEDI 1, 19 (14.4%) resided in SEDI 2, 23 (17.4%) in SEDI 3, 25 (18.9%) in SEDI 4, 18 (13.6%) in SEDI 5, and 20 (15.2%) in SEDI 6. The prevalence of significant fibrosis differed across destination groups, ranging from 3.7% among those who remained in SEDI 1 to 55.0% among those residing in SEDI 6; corresponding prevalences were 10.5% in SEDI 2, 17.4% in SEDI 3, 12.0% in SEDI 4, and 16.7% in SEDI 5. Overall differences across destination groups were significant in global tests (Fisher’s exact *P* < 0.001), and fibrosis stage distributions also differed significantly in the Kruskal–Wallis test (*P* < 0.001) (Fig. 7). When collapsed into a binary comparison, significant fibrosis was less frequent among participants who remained in SEDI 1 than among those who moved from SEDI 1 (1/27 [3.7%] vs 23/105 [21.9%]; Fisher’s exact *P* = 0.027), and fibrosis stage distributions likewise differed (Wilcoxon rank-sum *P* < 0.001).

### Conceptual directed acyclic graph (DAG) of the hypothesised access-related pathway

**Fig. 8:**
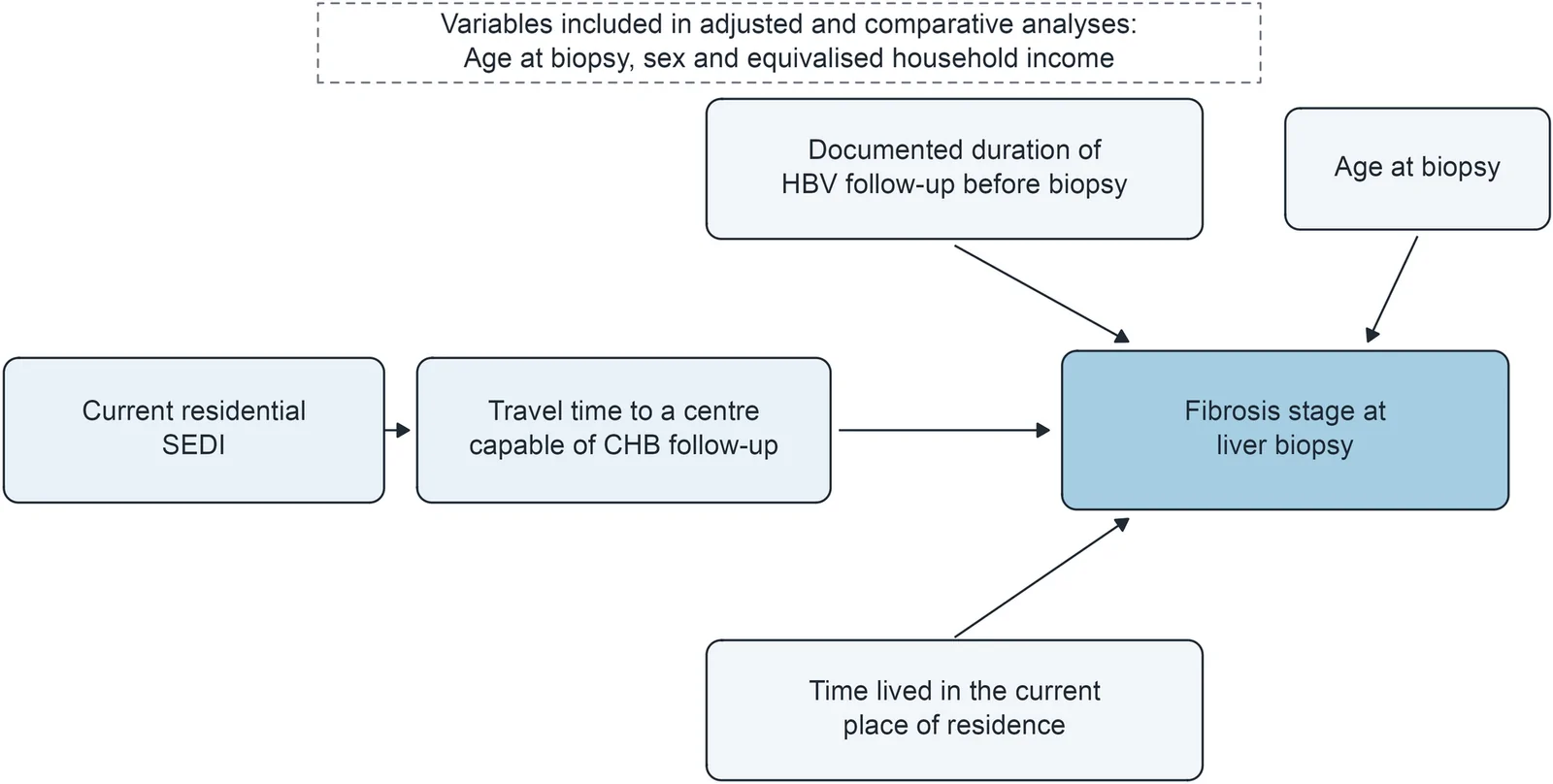
Conceptual directed acyclic graph of the hypothesised access-related pathway linking current residential SEDI to fibrosis stage at liver biopsy. The diagram summarises the hypothesised access-related pathway linking lower current residential SEDI to longer travel time to a centre capable of CHB follow-up and, both directly and through a measured pre-biopsy care marker, to more advanced fibrosis stage at liver biopsy. Documented duration of HBV follow-up before biopsy is shown as a distinct measured pathway variable associated with fibrosis severity rather than as a serial consequence of travel time. Age at biopsy and time lived in the current place of residence are shown as supporting contextual variables associated with fibrosis severity in the present study. Variables included in adjusted and comparative analyses are indicated separately above the main pathway. The figure is conceptual and does not imply that causal effects were identified in this observational study. Very light blue shading is used only to distinguish the primary exposure and outcome from measured pathway variables and supporting contextual variables; the colour scheme does not indicate effect size or direction. DAG directed acyclic graph; CHB chronic hepatitis B; HBV hepatitis B virus; SEDI socioeconomic development index category

A conceptual DAG was constructed to summarise the hypothesised access-related pathway linking current residential socioeconomic disadvantage to fibrosis severity at liver biopsy. In this framework, lower current residential SEDI is positioned upstream of greater travel burden and is linked, both directly and through measured pre-biopsy care markers, to higher fibrosis stage at histologic assessment. Documented duration of HBV follow-up before biopsy is presented as a distinct measured pathway variable associated with fibrosis severity, whereas age at biopsy and time lived in the current place of residence are shown as supporting contextual variables. Variables already incorporated in adjusted and comparative analyses are displayed separately from the main pathway (Fig. 8).

## Discussion

In this nationwide multicentre biopsy-based study of adults with CHB in Türkiye, liver fibrosis severity was associated more consistently with area-level socioeconomic disadvantage of the current place of residence than with birthplace socioeconomic context or most individual socioeconomic indicators. This pattern was evident in the primary ordinal regression analysis, remained marked in matched and overlap-weighted comparisons of the two current residential socioeconomic extremes, and was further reinforced in reciprocal birthplace-stratified subgroup analyses. Among participants born in SEDI 6, fibrosis burden was highest among those who remained in SEDI 6 and lower among those residing in more advantaged settings; conversely, among participants born in SEDI 1, fibrosis burden was lowest among those who remained in SEDI 1 and highest among those residing in SEDI 6. Longer travel time to a centre capable of CHB follow-up and shorter documented duration of HBV follow-up before biopsy were likewise associated with fibrosis severity. Taken together, these findings suggest that current residential socioeconomic context may be relevant not only to access to care, but also to the stage at which clinically meaningful liver disease is recognised, evaluated, and histologically staged.

Previous HBV research has increasingly linked socioeconomic disadvantage to barriers in vaccination, screening, linkage to care, specialist assessment, and retention in care [9, 17–19]. Area-level studies have likewise suggested that deprivation may be relevant to HBV-related risk and outcomes, including HBsAg positivity and hepatocellular carcinoma [10, 11]. Against that background, our findings extend the literature by showing that current residential disadvantage was also associated with biopsy-based fibrosis burden at presentation. This pattern is important because it suggests that disadvantage may be reflected not only in access to services or downstream complications, but also in the stage of liver disease at which patients come to histologic evaluation.

The existing literature also suggests that the CHB care continuum is highly sensitive to structural barriers, including affordability, navigation of care, and sustained engagement with services [9, 17–19]. In our study, longer travel time to a centre capable of CHB follow-up and shorter documented duration of HBV follow-up before biopsy were independently associated with higher fibrosis stage, alongside the persistent association between lower residential SEDI and more advanced fibrosis. Viewed together, these findings are consistent with an access-related pathway in which people living in more disadvantaged areas may reach specialist evaluation and biopsy under less favourable conditions, with more advanced fibrosis therefore observed at histologic assessment. This interpretation is summarised conceptually in Fig. 8. However, it should remain cautious, because referral thresholds, biopsy decision-making, and some interview-based measures were not captured directly, and the DAG was included as an interpretive rather than causal-identification framework.

Published CHB literature has more often examined birthplace in relation to prevalence, migration-associated disparities, or care engagement than considered birthplace and current residence together in relation to fibrosis severity [20–23]. In the present study, birthplace SEDI was not associated with fibrosis severity, whereas lower current residential SEDI and longer duration of residence in the current place were both associated with more advanced fibrosis. The subgroup analyses sharpened this contrast in both directions. Among participants born in SEDI 6, fibrosis burden remained highest among those who stayed in SEDI 6 and lower among those residing in more advantaged settings. Conversely, among participants born in SEDI 1, fibrosis burden was lowest among those who remained in SEDI 1 and highest among those residing in SEDI 6. This reciprocal pattern argues against a purely birthplace-based interpretation and instead suggests that fibrosis severity at biopsy may be more closely aligned with the social and material conditions under which patients are currently living and navigating follow-up, referral, and access to specialist assessment. These findings should not be over-interpreted as population-level migration effects, but they strengthen the central inference that current residential context carries more explanatory weight than birthplace alone.

Previous studies in socially disadvantaged and underserved HBV populations have also suggested that disadvantage often operates through structural and service-level barriers rather than through any single individual-level marker in isolation [9, 24, 25]. In our study, most individual socioeconomic indicators did not show independent associations with fibrosis severity, whereas area-level residential disadvantage showed a strong and consistent gradient. This pattern was further supported by exploratory sensitivity models restricted to participants residing in SEDI 1 or SEDI 6, in which the association between current residence in SEDI 6 and significant fibrosis remained strong after separate adjustment for equivalised household income, participant education, receipt of social assistance, and health insurance/social security coverage. If retained in the final multivariable model, the inverse association observed for social assistance should be interpreted cautiously, as it may reflect residual confounding, measurement limitations, or differential linkage to formal support structures rather than a protective effect in itself. Overall, these findings suggest that, in this clinically selected cohort, area-level residential disadvantage showed a stronger and more consistent association with fibrosis severity than the individual socioeconomic indicators available in this study. This should not be interpreted as a direct scale-equivalent comparison between SEDI and any single individual-level measure, but rather as evidence that current residential context captured dimensions of disadvantage not fully represented by individual income, education, insurance status, or social assistance.

The strengths of this study should be considered alongside these findings. Most published work in this field has relied on prevalence, care-cascade indicators, or later liver outcomes [9–11, 17–19]. By contrast, our study drew on a national multicentre cohort across 24 centres in Türkiye and combined biopsy-based histologic assessment with interviewer-administered socioeconomic data, allowing individual-level and area-level disadvantage to be examined within the same analytical framework. The consistency of the signal across the primary ordinal model, the extreme-group comparisons, and the reciprocal birthplace-stratified subgroup analyses increases confidence that the association with current residential context is not a single-model artefact. The separate consideration of birthplace and current residence also provided a more nuanced view of socioeconomic context than is usually available in CHB studies.

Several limitations should be acknowledged. First, the cohort was restricted to adults with CHB who presented to hospital-based care and underwent liver biopsy in routine clinical practice; therefore, findings may not be generalisable to the broader CHB population. Second, fibrosis severity was based on biopsy reports issued across participating centres, without retrospective central pathology review, and inter-pathologist variability could not be assessed. Third, interview-based socioeconomic indicators were collected after biopsy, with a median biopsy-to-interview interval of 60.0 months among participants with available interval data; these variables therefore reflected reported circumstances at the time of interview rather than necessarily at the time of histologic assessment. Because completion of the structured face-to-face interview was required for inclusion, selection bias may also have occurred if interview participation was related to socioeconomic position, continuity of follow-up, access to care, or fibrosis severity. Fourth, treatment history, virological activity, metabolic comorbidity, and centre-level practice variation were not incorporated into the present models. Comparisons between area-level SEDI and individual socioeconomic indicators should be interpreted cautiously because these measures capture different dimensions and scales of disadvantage. Accordingly, the RII analysis was used as an exploratory income-gradient analysis rather than as a definitive scale-equivalent comparison with SEDI, and exploratory sensitivity models incorporating individual socioeconomic indicators were intended to assess robustness after separate adjustment rather than to provide formal scale-equivalent comparisons. Propensity score matching and overlap weighting balanced age and sex only and should therefore be interpreted as supportive rather than definitive causal analyses, although regression-based sensitivity analyses with broader covariate adjustment yielded consistent results. Finally, this was an observational analysis, and residual confounding cannot be excluded.

Taken together, these findings suggest that current residential socioeconomic context may help identify adults with CHB who reach specialist evaluation and biopsy with a greater fibrosis burden. The results do not imply that residential disadvantage directly causes fibrosis progression, but they indicate that place-based disadvantage may capture access-related and structural conditions not fully reflected by individual socioeconomic indicators alone. From a public health perspective, this supports efforts to strengthen continuity of HBV follow-up, reduce travel burden, and improve timely access to specialist assessment in socioeconomically disadvantaged settings. Future studies incorporating virological, metabolic, treatment-related, and centre-level data are needed to clarify the mechanisms underlying these associations.

## Conclusions

Liver fibrosis severity in adults with CHB was associated more consistently with current residential socioeconomic disadvantage than with birthplace socioeconomic context or most individual socioeconomic indicators. This pattern was observed across multiple analytical approaches, including reciprocal birthplace-stratified subgroup analyses, and suggests that current residential context may be closely linked to fibrosis burden at biopsy. These findings support a place-based interpretation of inequality in CHB care and point to the need for earlier specialist evaluation and stronger continuity of follow-up in socioeconomically disadvantaged settings.

## Supplementary Information

Additional file 1.

Additional file 2.

Additional file 3.

Additional file 4.

Additional file 5.

Additional file 6.

Additional file 7.

## Data Availability

No datasets were generated or analysed during the current study.

## Abbreviations

CHB: Chronic hepatitis B
*CI*: Confidence interval
DAG: Directed acyclic graph
ESS: Effective sample size
FIB-4: Fibrosis-4 index
HBsAg: Hepatitis B surface antigen
HBV: Hepatitis B virus
HCV: Hepatitis C virus
HDV: Hepatitis D virus
HIV: Human immunodeficiency virus
*OR*: Odds ratio
RII: Relative index of inequality
SD: Standard deviation
SEDI: Socioeconomic development index
STROBE: Strengthening the Reporting of Observational Studies in Epidemiology
