# Supplementary material for: Liver fibrosis severity in chronic hepatitis B is associated with area-level rather than individual socioeconomic disadvantage: a nationwide multicentre study in Türkiye
Source: Infect Dis Poverty. 2026 Sep 3;15:96. doi: 10.1186/s40249-026-01497-8 (PMC13540940; doi:10.1186/s40249-026-01497-8)
Supplement: Supplementary file 1 [file 40249_2026_1497_MOESM4_ESM.pdf]

Liver Fibrosis in Chronic Hepatitis B: A Social Epidemiological Perspective

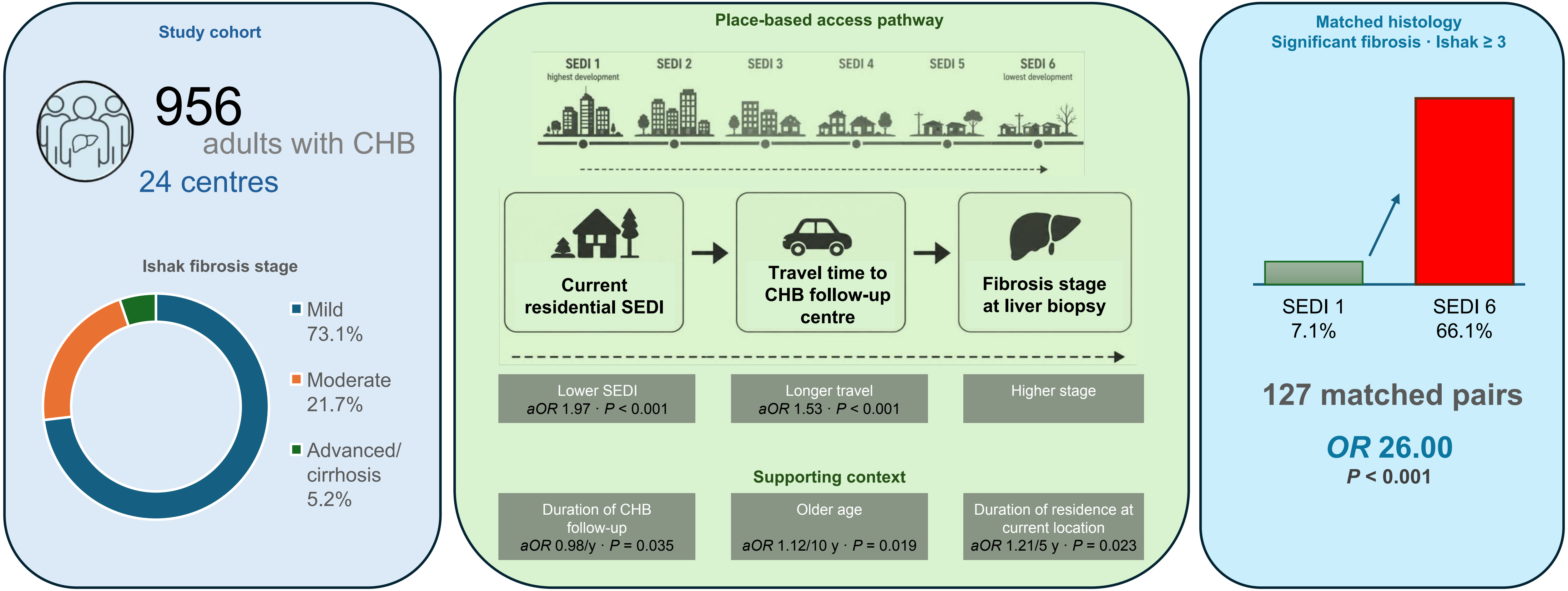

Current residential disadvantage showed the clearest association with fibrosis severity, beyond birthplace and most individual socioeconomic indicators.
