# Supplementary material for: Liver fibrosis severity in chronic hepatitis B is associated with area-level rather than individual socioeconomic disadvantage: a nationwide multicentre study in Türkiye
Source: Infect Dis Poverty. 2026 Sep 3;15:96. doi: 10.1186/s40249-026-01497-8 (PMC13540940; doi:10.1186/s40249-026-01497-8)
Supplement: Supplementary file 2 [file 40249_2026_1497_MOESM5_ESM.docx]

Supplementary Table S1. Sensitivity analysis of the association between current residential SEDI category and significant liver fibrosis after broader covariate adjustment

| **Model** | **Covariates included** | ***n*** | **Events, *n*** | **SEDI 1, *n*** | **SEDI 6, *n*** | **Adjusted *OR* (95% *CI*)** | ***P* value** |
| --- | --- | --- | --- | --- | --- | --- | --- |
| Model 1 | Age at biopsy and sex | 320 | 126 | 144 | 176 | 28.83 (13.50–61.57) | < 0.001 |
| Model 2 | Model 1 plus individual socioeconomic variables: equivalised household income, educational level, health insurance/social security status, and receipt of social assistance | 296 | 118 | 130 | 166 | 28.17 (12.65–62.69) | < 0.001 |
| Model 3 | Model 2 plus access- and follow-up-related variables: duration of hepatitis B virus follow-up before biopsy, follow-up interval, and travel time to the nearest healthcare facility | 293 | 115 | 130 | 163 | 18.98 (8.43–42.72) | < 0.001 |

Significant liver fibrosis was defined as Ishak fibrosis stage ≥ 3. Analyses were restricted to participants residing in the two extreme current residential SEDI categories, SEDI 1 and SEDI 6. The adjusted odds ratios compare current residence in SEDI 6 with current residence in SEDI 1.

Model 1 corresponds to the demographic covariate set used in the propensity score matching and overlap-weighting analyses. Model 2 evaluates whether the association persists after broader adjustment for individual socioeconomic variables. Model 3 additionally includes access- and follow-up-related variables; these variables may plausibly lie on the hypothesised pathway between residential socioeconomic disadvantage and fibrosis severity and were therefore interpreted as exploratory/pathway-adjusted covariates rather than as the primary confounding-control set.

***Abbreviations:*** *CI, confidence interval; OR, odds ratio; SEDI, socioeconomic development index.*
