# Supplementary material for: Liver fibrosis severity in chronic hepatitis B is associated with area-level rather than individual socioeconomic disadvantage: a nationwide multicentre study in Türkiye
Source: Infect Dis Poverty. 2026 Sep 3;15:96. doi: 10.1186/s40249-026-01497-8 (PMC13540940; doi:10.1186/s40249-026-01497-8)
Supplement: Supplementary file 3 [file 40249_2026_1497_MOESM6_ESM.docx]

**Supplementary Table S2.** Exploratory sensitivity models of the association between current residential SEDI category and significant liver fibrosis after separate adjustment for individual SES indicators

| **Model** | **Added individual SES indicator** | **Comparison** | ***n*** | **Events, *n*** | **Adjusted *OR* (95% *CI*) for SEDI 6 versus SEDI 1** | ***P* value for SEDI** | **Adjusted *OR* (95% *CI*) for individual SES comparison** | ***P* value for individual SES comparison** |
| --- | --- | --- | --- | --- | --- | --- | --- | --- |
| Model A | Equivalised household income | Per doubling of equivalised household income | 296 | 118 | 24.89 (11.54–53.68) | < 0.001 | 1.07 (0.81–1.41) | 0.641 |
| Model B | Participant educational level | Middle versus high education | 320 | 126 | 29.87 (13.83–64.54) | < 0.001 | 1.04 (0.49–2.23) | 0.917 |
| Model B | Participant educational level | Low versus high education | 320 | 126 | 29.87 (13.83–64.54) | < 0.001 | 1.45 (0.64–3.28) | 0.376 |
| Model C | Receipt of social assistance | Yes versus no | 320 | 126 | 29.97 (13.93–64.45) | < 0.001 | 0.25 (0.07–0.97) | 0.046 |
| Model D | Health insurance or social security coverage | Non-standard or limited coverage versus standard or private insurance | 320 | 126 | 29.18 (13.60–62.61) | < 0.001 | 2.66 (0.62–11.40) | 0.188 |

Significant liver fibrosis was defined as Ishak fibrosis stage ≥ 3. Analyses were restricted to participants residing in the two extreme current residential SEDI categories, SEDI 1 and SEDI 6. Each model included current residential SEDI category, age at biopsy, sex, and one individual socioeconomic indicator entered separately. The adjusted odds ratio for SEDI compares current residence in SEDI 6 with current residence in SEDI 1. Age at biopsy was modelled per 10-year increase, and sex was included as a binary covariate. Equivalised household income was calculated by dividing household income by the square root of household size and was modelled on the log2 scale; therefore, the income estimate represents the adjusted odds ratio per doubling of equivalised household income. Education was categorised as high, middle, and low, with high education as the reference category. Health insurance or social security coverage was collapsed as standard or private insurance versus non-standard or limited coverage because of sparse cell counts in the no-insurance category. Models used complete-case data for the variables included in each model. These analyses were exploratory and were intended to evaluate whether the association between current residential SEDI and significant fibrosis was materially changed after separate adjustment for individual socioeconomic indicators; they were not intended as scale-equivalent comparisons between area-level and individual-level socioeconomic measures.

***Abbreviations:*** *CI, confidence interval; OR, odds ratio; SEDI, socioeconomic development index; SES, socioeconomic status.*
